# Erosive tooth wear among non-institutionalised older adults in Hong Kong: a cross-sectional study

**DOI:** 10.1186/s12903-023-03835-w

**Published:** 2024-01-09

**Authors:** Darren Dhananthat Chawhuaveang, Duangporn Duangthip, Alice Kit-Ying Chan, Samantha Kar-Yan Li, Chun-Hung Chu, Ollie Yiru Yu

**Affiliations:** 1 Faculty of Dentistry, The University of Hong Kong, Hong Kong SAR, China; 2 Department of Restorative Dental Sciences, Faculty of Dentistry, Prince Philip Dental Hospital, The University of Hong Kong 3B12, 34 Hospital Road, Hong Kong, 999077 China

**Keywords:** Erosive tooth wear, Tooth erosion, Epidemiology, Cross-sectional study, Older adults

## Abstract

**Background:**

Non-institutionalised older adults is the majority of older adults in Hong Kong. The study aimed to examine erosive tooth wear (ETW) and its association with dental conditions and oral hygiene habits among non-institutionalised older adults in Hong Kong.

**Methods:**

This cross-sectional study recruited dentate adults aged 60 or above from nine elderly daycare centres in the five main districts of Hong Kong. The study consists of a questionnaire survey and a clinical examination. A researcher used a questionnaire to collected the participants’ demographic information, oral hygiene habits such as toothbrushing habits and dental visit behaviour. A calibrated examiner performed an oral examination in the daycare elderly centre to assess the ETW using basic erosive wear (BEWE) criteria. Oral hygiene was recorded using visible plaque index. Prosthetic status was recorded using the World Health Organization criteria. Logistic regression was used to examine the correlation between ETW and the dental conditions and oral hygiene habits.

**Results:**

This study recruited 433 dentate adults and 333 adults were female (77%). Their age ranged from 60 to 99 years and their mean age was 74 years (SD = 7). They all had ETW (BEWE > 0). Over half of them (57%) had BEWE score of 3, indicating severe ETW. Analysis showed increasing age (OR = 1.030, *p* = 0.029) and older adults with untreated dental caries had higher odds (OR = 1.822, *p* = 0.002) of presenting severe ETW. No other associations were found between the ETW and the factors studied.

**Conclusion:**

Hong Kong non-institutionalised older adults aged 60 or above had ETW and more than half of them had severe ETW. Increasing age and having untreated dental caries were associated with severe ETW.

## Background

The ageing of the population is a global challenge. The United Nations estimated the number of older adults worldwide will double in 2050 [1]. Hong Kong is also facing the problem. With the increased life expectancy of the residents and the declining birth rate [2, 3], the demographic structure of the population of Hong Kong has shifted with an enlarged proportion of older adults [2, 3]. According to the Census and Statistics Department report in 2022, there were 2.2 million residents aged 60 years or over, which consists of 30% of the 7.5 million Hong Kong population [3]. The number is expected to reach 2.58 million by 2069 [3].

Population ageing has a significant impact on the health care system. It leads to an increased demand for healthcare services, higher healthcare costs, and a shortage of healthcare professionals and long-term care facilities [2, 4]. It places a significant burden on healthcare systems and public finances [2, 4]. It is essential to develop strategies to address the impact of ageing on the healthcare system, which requires a deep understanding of the population. Therefore, epidemiological information on the health status of the population should be obtained to develop long-term plans and oral health policies [5].

Oral diseases have a detrimental impact on both the oral and general health of older adults, significantly compromising their quality of life [6, 7]. Good oral health is essential for the ability to taste, chew, and swallow, making it crucial for the overall well-being of older individuals [6, 8, 9]. Oral diseases can cause pain and anxiety, hinder chewing and eating abilities, impede nutritional intake, and pose a threat to the general health of older adults [4, 6, 7].

However, oral disease is prevalent and affects a significant proportion of older adults [10]. The prevalence of oral diseases affecting dental hard tissue is on the rise among older adults [7–10]. As the population’s lifespan increases, dental hard tissue in the oral cavity is required to function for longer periods, making it more susceptible to diseases [11]. Erosive tooth wear (ETW) is one of the prevalent dental hard tissue diseases affecting older adults [12].

Dental erosion refers to the dissolution of tooth surfaces due to chemical processes from non-bacterial acids [13]. ETW involves the loss of softened tooth surfaces caused by mechanical forces [11, 13]. For instance, wearing dental prosthesis may increase the risk of ETW. The static and slide friction forces from denture base and clasp of the removable partial denture may wear the contact area of the tooth [14]. The frequency of placing and removing the denture from the mouth may also affect the progress of ETW [15]. Moreover, fixed prostheses may increase the risk of ETW on antagonist teeth [16]. Severe ETW can result in the loss of tooth structure, poor aesthetics, and diminished chewing ability [17, 18]. Moreover, severe ETW is challenging to treat and can negatively impact oral health-related quality of life [4, 12]. The prevalence of ETW in children and adults is reported to range from 7.2 to 95.0% in the literature [18, 19]. Studies have been published on the prevalence of ETW in children, adolescents, and young adult populations in Hong Kong, with reported rates ranging from 14.9 to 75% [12, 17, 19]. Our literature search revealed no data on the ETW among older adults in Hong Kong. However, other studies on the oral health status of the older adult population in Hong Kong focused on institutionalised older adults, especially in the chronic health condition group (5, 16, 20). Therefore, data from non-institutionalised older adults in Hong Kong, which is the majority of older adults [2, 3], is warranted.

Consequently, the objective of this study was to examine ETW and its associations with dental conditions and oral hygiene habits among non-institutionalised older adults aged 60 or above in Hong Kong.

## Methods

This cross-sectional observational study was approved by the Institutional Review Board of the University of Hong Kong/Hospital Authority Hong Kong West Cluster (IRB UW 21–316). This study was conducted in 2021–2023. The report in this study was in accordance with the Strengthening the Reporting of Observational Studies in Epidemiology (STROBE) statement [20].

### Sample size calculation

Sample size calculation was performed by G*Power software Version 3.1 and based on the prevalence report in older adults, which reported 62% of older adults had ETW [21]. Sample size of 412 participants would have 80% power to detect an odds ratio of 1.8 based on 60% of the participants having ETW at the 0.05 significant level while assuming the allocation ratio was equal. Assuming the response rate was 90%, the number of older adults to be invited would be at least 457.

### Recruitment of participants

Hong Kong has 11 District Elderly Community Centres (DECCs) with a total of 213 elderly daycare centres, and all Hong Kong older adults aged 60 or above must enrol in DECCs which are organized by the Social Welfare Department. The target population were recruited from 5 DECCs from 9 elderly daycare centres due to the permission of Hong Kong government policy during the COVID-19 outbreak. Five DECCs were as follows, Kowloon City and Yau Tsim Mong Districts, Sham Shui Po District, Yuen Long District, Tsuen Wan and Kwai Tsing Districts, and Tuen Mun District. All eligible participants in each daycare centre were invited to the study through invitation letter. Written informed consent was obtained before the clinical examination. The inclusion criteria included dentate older adults aged 60 or above who were cooperative for intraoral examination. The exclusion criteria were participants aged less than 60, complete edentulism, and older adults who had severe systemic diseases such as poorly controlled diabetes mellitus or hypertension, end-stage renal disease, history of acute myocardial infarction or cerebrovascular accident or transient ischemic attack [22]. Older adults who fulfilled the eligibility criteria were enrolled in the study.

### Questionnaire survey

The questionnaire was designed and validated according to the study model reported by previous studies [11, 23–27]. The questionnaire is shown in Table 1. The questionnaire included questions regarding personal information (age and gender) and oral hygiene habits such as toothbrushing habits and dental visit behaviour. Before the clinical examination, participants were asked to complete the questionnaire on-site. An investigator (D.D.C) checked the completed questionnaire when it was returned.

**Table 1:** Questionnaire used in this study

1. Age……………………					
2. Gender					
☐ Male	☐ Female				
3. What is the daily frequency of your performing tooth brushing?					
☐ 1 or less	☐ 2	☐ 3 or more			
4. Do you use a toothbrush for cleaning your teeth?					
☐ Yes	☐ No				
5. Do you use an interdental brush for cleaning your teeth?					
☐ Yes	☐ No				
6. Do you use a toothpick for cleaning your teeth?					
☐ Yes	☐ No				
7. Do you wear dentures?					
☐ Yes	☐ No				
8. When is your last dental visit?					
☐ Less than 1 year		☐ 1–3 years		☐ More than 3 years	

### Clinical examination

The clinical examination was conducted at elderly daycare centres by one of two well-trained examiners using non-invasive and painless methods. The clinical examination followed the basic procedures recommended by the World Health Organization (WHO), 2013 [28]. No radiograph examination was conducted during the clinical assessment, and no lifestyle or dietary restrictions were required before or after the oral health survey. Both examiners were trained two weeks before the survey to calibrate the oral health assessment. The intra and inter-examiner calibration between the two examiners exceeded 0.70 by the end of training. One-tenth of the participants at each centre were re-examined to determine the inter-examiner agreement. The duplicated clinical examination was performed at least 30 minutes after the first examination.

The clinical examination was conducted using a disposable dental mirror with an attached intraoral light-emitting diode light and a ball-ended WHO probe [12]. Cotton rolls were used for moisture control during the clinical examination. Data recording was carried out by two trained dental assistants. The oral health assessment of participants encompassed prosthetic status, percentage of visible plaque index (VPI), and ETW status. ETW status was recorded with a BEWE index [29]. After the clinical examination, participants received a document detailing their oral health status. Older adults with dental problems requiring treatment were advised to schedule dental treatment appointments at nearby hospitals.

The prosthetic status was examined for both the upper and lower arches, with six conditions: score 0 = no prostheses, score 1 = one bridge, score 2 = more than one bridge, score 3 = partial denture, score 4 = both bridge and partial denture and score 5 = complete denture.

Oral hygiene status was determined using the Visible Plaque Index (VPI) on the buccal and lingual surfaces of index teeth (tooth number 17/16, 11, 26/27, 36/37, 31, 46/47). The visible plaque was as follows: not presence = score 0, presence = score 1 and missing tooth = score 9. The VPI score was calculated as the ratio of the number of surfaces with plaque to the total number of examined surfaces.

The basic erosive wear examination (BEWE) score included four classifications: score 0 = no erosive tooth wear, score 1 = initial loss of enamel surface, score 2 = distinct defect of hard tissue loss less than 50% of the surface area, score 3 = distinct defect of hard tissue loss more than 50% of the surface area [29]. The highest BEWE score on the tooth surface in each sextant represents the ETW status of the entire sextant [12, 29].

### Data analysis

The data were recorded on a charting form specifically designed for oral health examinations of elderly individuals in Hong Kong. The data from the questionnaire and clinical examination were input into Microsoft Excel. Data analysis was performed using IBM SPSS Statistics for Windows, Version 28.0. Intra and inter-examiner agreement was assessed with Cohen’s kappa coefficient based on tooth level. A chi-square test was performed to analyse the differences in the presence of severe ETW (BEWE = 3) according to the distribution of older adult’s demographic characteristics (age and gender), oral health status (number of remaining teeth and denture prostheses), oral hygiene habits (frequency of perform toothbrushing, types of mechanical oral hygiene aids, untreated dental caries, and dental visit experience). Owing to the asymmetric data distribution of age and VPI (by Shapiro-Wilk test), a non-parametric test; the Mann-Whitney test was performed to investigate the association between severe ETW (binary variable using cut-off value BEWE = 3) and VPI and age variables. All the variables were evaluated using a logistic regression model to determine the risk factors of severe ETW in the participants. A backward stepwise was adopted to remove the variables that were not statistically significant. The final model included only statistically significant variables. The level of significance was set at 0.05.

## Results

**Fig. 1:**
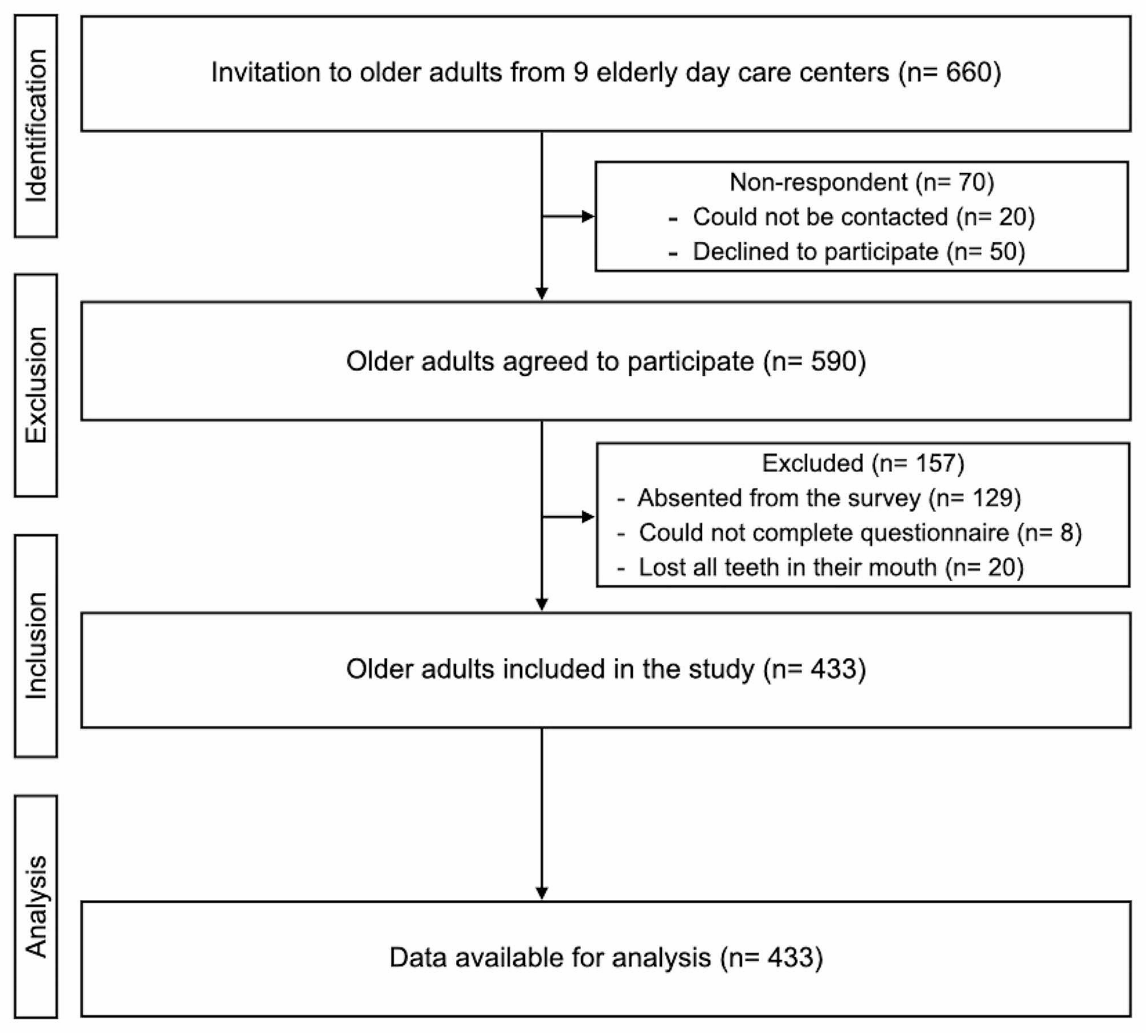
Flow chart of the recruitment of older adults in this study

The flow chart of the recruitment of older adults is displayed in Fig. 1. A total of 660 older adults were invited to join the study. 590 older adults agreed to join and 157 older adults were excluded because they absented the survey, could not complete the questionnaire, and could not meet the inclusion criteria. Thus, 433 older adults from 9 elderly daycare centres were examined. The response rate was 73%. Among the 433 participants, approximately three-quarters of the participants were female (n = 333, 76.90%). The mean age of all participants (± SD) was 74 ± 7 years. The kappa value of inter-examiner agreement for the assessment of BEWE was 0.81. Intra-examiner agreement of two examiners were 0.83 and 0.85.

### Prosthetic status

The prosthetic status of the upper and lower arch is shown in Table 2. Approximately half of older adults did not have prostheses. The most common type of upper and lower prostheses was bridge, following by partial denture and complete denture.

**Table 2:** Prosthetic status

Dental arch	No Prostheses	1 Bridge	More than 1 bridge	Partial denture	Both bridge and partial denture	Complete denture
Upper	52.89% (229)	15.70% (68)	10.16% (44)	14.32% (62)	1.85% (8)	5.08% (22)
Lower	56.58% (245)	15.24% (66)	8.78% (38)	13.86% (60)	1.85% (8)	3.69% (16)

### Erosive tooth wear status

The presence and severity of ETW in Hong Kong older adults, stratified by gender are described in Table 3. BEWE score 0 was detected in none of the participants. ETW (BEWE > 0) presented in 100% of the participants, while severe ETW (BEWE = 3) presented in 56.81% of the participants. The mean age of participants who had severe ETW was 74.3 ± 7.6 years. More than half of males (65%) and females (54.4%) had severe ETW (BEWE = 3). The mean BEWE scores per sextant for males and females were 2.1 ± 0.5 and 2.0 ± 0.5, respectively. The distribution of the severity of ETW in the six sextants is presented in Table 4. The lower anterior teeth had a higher presence and severity of ETW than the other areas.

**Table 3:** Presence and severity of erosive tooth wear in Hong Kong older adults

Gender (n)	Highest BEWE score					BEWE score per sextant Mean (SD)
	0 (No ETW)		1 (Initial loss)	2 (Distinct loss < 50%)	3 (Distinct loss > 50%)	
Male (100)	0 (0%)		3 (3.0%)	32 (32.0%)	65 (65.0%)	2.1 (0.5)
Female (333)	0 (0%)		20 (6.0%)	132 (39.6%)	181 (54.4%)	2.0 (0.5)
All (433)	0 (0%)		23 (5.31%)	164 (37.88%)	246 (56.81%)	2.0 (0.5)

**Table 4:** Distribution of the severity of erosive tooth wear in each sextant

Sextant	BEWE score				
	0 (No ETW)	1 (Initial loss)	2 (Distinct loss < 50%)	3 (Distinct loss > 50%)	No tooth to evaluate
1: Upper right posterior teeth	3.00% (13)	25.17% (109)	44.11% (191)	8.55% (37)	19.17% (83)
2: Upper anterior teeth	2.08% (9)	15.70% (68)	59.35% (257)	11.09% (48)	11.78% (51)
3: Upper left posterior teeth	1.15% (5)	18.71% (81)	53.12% (230)	8.31% (36)	18.71% (81)
4: Lower right posterior teeth	0.69% (3)	17.09% (74)	55.66% (241)	7.85% (34)	18.71% (81)
5: Lower anterior teeth	0.23% (1)	9.47% (41)	38.11% (165)	44.57% (193)	7.62% (33)
6: Lower left posterior teeth	0.46% (2)	14.78% (64)	53.58% (232)	9.70% (42)	21.48% (93)

The bivariate analysis (Table 5) showed that frequency of performing tooth brushing (1/2/≥ 3; *p* = 0.032), untreated dental caries (yes/no; *p* = 0.002), and age (*p* = 0.019) were associated with the presence of severe ETW (BEWE = 3) with statistical significance. However, no statistically significant differences were found in the presence of severe ETW (BEWE = 3) among gender (male/female; *p* = 0.066), use of toothbrush (yes/no; *p* = 1.000), use of interdental brush (yes/no; *p* = 0.290), use of toothpick (yes/no; *p* = 0.687), tooth condition (< 20 teeth/≥ 20 teeth; *p* = 0.201), having fixed prostheses (yes/no; *p* = 0.275), wearing removable prostheses (yes/no; *p* = 1.000), dental visit experience (less than one year/1–3 years ago/more than 3 years ago; *p* = 0.264), and VPI (*p* = 0.455).

The results of the logistic regression are displayed in Table 6. The logistic regression analysis showed that age and having untreated dental caries were statistically associated with the presence of severe ETW, whereas other variables were not statistically significant. The final model examined by backward stepwise binary regression generated the same results as those obtained from the forward stepwise procedure. Participants with increasing age and having untreated dental caries had a higher chance for severe ETW. Increasing age had a significantly higher chance (OR:1.030, 95% C.I: 1.003–1.059) of having severe ETW. Older adults who had untreated dental caries had a higher chance of severe ETW compared to older adults who did not have untreated dental caries (OR: 1.822, 95% C.I: 1.237–2.684).

**Table 5:** Severe erosive tooth wear according to variables studied

Variable (n)	BEWE = 3 (n, %)	*p* value
Gender		
Male (100) Female (333)	65 (65.0%) 181 (54.4%)	0.066
Frequency of performing tooth brushing		
1 or less (55) 2 (343) ≥ 3 (35)	37 (67.3%) 184 (53.6%) 25 (71.4%)	0.032
Type of mechanical oral hygiene practice		
1. Toothbrush		
Yes (428) No (5)	243 (56.8%) 3 (60.0%)	1.000
2. Interdental brush		
Yes (69) No (364)	35 (50.7%) 211 (58.0%)	0.290
3. Toothpick		
Yes (158) No (275)	92 (58.2%) 154 (56.0%)	0.687
Tooth condition		
< 20 teeth (254) ≥ 20 teeth (179)	151 (59.4%) 95 (53.1%)	0.201
Untreated dental caries		
Yes (234) No (199)	149 (63.7%) 97 (48.7%)	0.002
Fixed prostheses		
Yes (168) No (265)	101 (60.1%) 145 (54.7%)	0.275
Removable prostheses		
Yes (116) No (317)	66 (56.9%) 180 (56.8%)	1.000
Dental visit experience		
more than 3 years ago (171) 1–3 years ago (122) Less than one year (140)	103 (60.2%) 62 (50.8%) 81 (57.9%)	0.264
Age		0.019
VPI		0.455

**Table 6:** Severe erosive tooth wear according to significant variables

Variable (n)	Odd ratio	95% CI	*p* value
Age	1.030	1.003-1.059	0.029
Untreated dental caries Yes No*	1.822	1.237-2.684	0.002

* = Reference group; CI = Confidence interval

## Discussion

This study was the first epidemiological survey in Hong Kong to report ETW among non-institutionalised older adults aged 60 or above. The population aged 60 and above in Hong Kong is 2.19 million (30% of the population), which is the majority of the Hong Kong population [3]. In addition, the common retirement age of civil servants, non-governmental organisations employees, and government-aid organisations staff in Hong Kong is 60 years old [30]. The data collected from this study provided valuable insights into the presence of ETW in older adults in Hong Kong, which poses significant challenges to their oral health and has long-term implications on their oral and general health [31, 32]. In addition, this study identified independent variables related to oral hygiene habits, which were associated with the presence of severe ETW. These findings may encourage dental professionals to be more vigilant in detecting ETW during regular check-ups for older adults. The data would be helpful for the government and dental professions to plan for community-based preventive measurements for older adults at retirement age. Moreover, it can serve as baseline data for future full-scale population surveys on ETW in older adults in Hong Kong in the future.

The presence of ETW (BEWE > 0) was 100%, while the presence of severe ETW (BEWE = 3) was 56.81% among older adults aged 60 or above. The presence of ETW in the current study was consistent with previous findings in Wuhan City, P.R. China, among Chinese adults aged 50–74 [11]. It is higher than the 40% prevalence observed in Munich, Germany, among older adults aged 65–74 [21, 33] and higher than the 61.9% prevalence in Kibbutz, Israel, among older adults ages 55–60 [34]. The differences in the prevalence of ETW among older adults might be attributed to variations in ETW indices, sample sizes, and geographical factors [19, 21].

In this study, all oral examinations except the ETW assessment followed the guidelines of oral health surveys by the World Health Organization [28]. To date, there is no consensus on the best method for ETW assessment [11, 12]. The basic erosive wear examination (BEWE) is a convenient and time-saving method with high sensitivity and specificity for detecting moderate to severe ETW lesions [11, 35]. This study utilised the BEWE index by Bartlett et al. [29]. The presence of ETW was determined using a BEWE score greater than 0, resulting in 100% of older adults in this study having ETW. However, for the statistical analysis of independent variables related to severe ETW presence, a BEWE score of 3 was used, as no participants were free from tooth wear. This score has high sensitivity and specificity for measuring tooth surface loss in a clinical setting [35], making it a suitable dependent variable for this study [35].

This study found that gender was not statistically associated with the presence of severe BEWE (*P* = 0.066), which is inconsistent with a previous study that reported males had a higher prevalence of ETW than females [21]. This study found no statistically significant association between severe ETW and frequency of toothbrushing, which is consistent with a previous study [11]. Increased age showed a positive relationship with a higher chance of having severe ETW among older adults, supporting the previous study that mentioned an age-dependent increase in ETW prevalence [11, 21]. Similarly, the pathological finding of ETW lesions progresses with time [12]. Moreover, older adults with untreated dental caries in their mouths had a significantly higher chance (1.822 times) of severe ETW. This finding was consistent with a previous study [36]. The possible reasons can be drawn on this issue that their perception of the need for dental care remains poor, physically unfit, deny dental treatment or more frequent acidic drinks or foods [4, 36].

This study used a closed-ended questionnaire to gather information on dental habits and practices. However, information on dietary habits, such as the frequency and quantity of acidic drinks, beverages or medication daily by older adults, was not collected. The Thematic Household Survey Report of Hong Kong in 2009 reported that 40% of older adults experienced difficulty remembering events that occurred a few minutes prior [4]. Completing complex questionnaires could be challenging and time-consuming for older adults [37]. Moreover, asking them to recall certain details may lead to inaccurate results due to age-related declines in recall ability [37].

This study has some limitations. This cross-sectional observational study was performed during the COVID-19 outbreak when many elderly daycare centres in Hong Kong imposed visiting restrictions. Due to limited resources and time constraints at each centre, we employed a consecutive sampling method instead of a probability sampling method. This approach is considered the best among non-probability sampling methods, as it is simple and efficient, allowing for the inclusion of all potential participants [38]. However, this method may contain a potential risk of selection bias [17]. To minimise the sampling bias, we recruited participants from nine elderly daycare centres in different areas of Hong Kong and ensured no overlap among participants. In addition, according to the results of this study, more than three-quarters of the participants were female. This was in accordance with the previous report that elderly females were more likely to engage in social activities than elderly males [38]. Hence, the results of this study should be interpreted with caution because of these limitations.

## Conclusion

All Non-institutionalised older adults in Hong Kong had ETW. Increasing age and having untreated dental caries were associated with severe ETW. No associations were found between the severe ETW, their dental conditions, and oral hygiene habits.

## Data Availability

The datasets used for this study are available from the first author on reasonable request.

## Abbreviations

ETW: Erosive tooth wear
BEWE: basic erosive wear
SD: Standard deviation
OR: Odd ratio
STROBE: Strengthening the Reporting of Observational Studies in Epidemiology
DECC: District Elderly Community Centres
WHO: World Health Organization
VPI: Visible plaque index
CI: Confidence interval
